# Incidence and Epidemiology of Hospitalized Influenza Cases in Rural Thailand during the Influenza A (H1N1)pdm09 Pandemic, 2009–2010

**DOI:** 10.1371/journal.pone.0048609

**Published:** 2012-11-06

**Authors:** Henry C. Baggett, Malinee Chittaganpitch, Somsak Thamthitiwat, Prabda Prapasiri, Sathapana Naorat, Pongpun Sawatwong, Darunee Ditsungnoen, Sonja J. Olsen, James M. Simmerman, Prasong Srisaengchai, Somrak Chantra, Leonard F. Peruski, Pathom Sawanpanyalert, Susan A. Maloney, Pasakorn Akarasewi

**Affiliations:** 1 International Emerging Infections Program, Thailand Ministry of Public Health (MOPH) – U.S. Centers for Disease Control and Prevention Collaboration, Nonthaburi, Thailand; 2 Global Disease Detection Branch, Global Disease Detection and Emergency Response Division, Centers for Disease Control and Prevention, Atlanta, Georgia, United States of America; 3 National Institute of Health, MOPH, Nonthaburi, Thailand; 4 Nakhon Phanom Provincial Health Office, Nakhon Phanom, Thailand; 5 Influenza Division, Centers for Disease Control and Prevention, Atlanta, Georgia, United States of America; 6 Sa Kaeo Provincial Health Office, MOPH, Sa Kaeo, Thailand; 7 Bureau of Epidemiology, MOPH, Nonthaburi, Thailand; Harvard School of Public Health, United States of America

## Abstract

**Background:**

Data on the burden of the 2009 influenza pandemic in Asia are limited. Influenza A(H1N1)pdm09 was first reported in Thailand in May 2009. We assessed incidence and epidemiology of influenza-associated hospitalizations during 2009–2010.

**Methods:**

We conducted active, population-based surveillance for hospitalized cases of acute lower respiratory infection (ALRI) in all 20 hospitals in two rural provinces. ALRI patients were sampled 1∶2 for participation in an etiology study in which nasopharyngeal swabs were collected for influenza virus testing by PCR.

**Results:**

Of 7,207 patients tested, 902 (12.5%) were influenza-positive, including 190 (7.8%) of 2,436 children aged <5 years; 86% were influenza A virus (46% A(H1N1)pdm09, 30% H3N2, 6.5% H1N1, 3.5% not subtyped) and 13% were influenza B virus. Cases of influenza A(H1N1)pdm09 first peaked in August 2009 when 17% of tested patients were positive. Subsequent peaks during 2009 and 2010 represented a mix of influenza A(H1N1)pdm09, H3N2, and influenza B viruses. The estimated annual incidence of hospitalized influenza cases was 136 per 100,000, highest in ages <5 years (477 per 100,000) and >75 years (407 per 100,000). The incidence of influenza A(H1N1)pdm09 was 62 per 100,000 (214 per 100,000 in children <5 years). Eleven influenza-infected patients required mechanical ventilation, and four patients died, all adults with influenza A(H1N1)pdm09 (1) or H3N2 (3).

**Conclusions:**

Influenza-associated hospitalization rates in Thailand during 2009–10 were substantial and exceeded rates described in western countries. Influenza A(H1N1)pdm09 predominated, but H3N2 also caused notable morbidity. Expanded influenza vaccination coverage could have considerable public health impact, especially in young children.

## Introduction

Increasing evidence supports influenza viruses as an important cause of disease in tropical and subtropical regions, particularly in young children and older adults [1], [2], [3], [4], [5]. Most studies from tropical Asia have documented year-round influenza virus circulation with fairly consistent seasonal peaks. However, the contribution of influenza viruses to severe disease in this setting, including pneumonia and especially among children, continues to be underappreciated. While influenza vaccination is universally recommended to all persons 6 months of age and older in the United States [6], vaccination guidelines are either non-existent or far more limited in most tropical countries, where the burden may be as great or greater.

The first cases of influenza A(H1N1)pdm09 infection were detected in the United States in April 2009. The ensuing pandemic resulted in heightened influenza surveillance worldwide. However, few surveillance systems included established catchment populations, meaning that population-based estimates of influenza A(H1N1)pdm09 incidence are limited, especially in tropical and resource limited settings. Robust incidence data are needed to estimate the burden of the 2009 pandemic and are important to inform policy discussions about prevention and control strategies for influenza in general.

The first cases of influenza A(H1N1)pdm09 were reported in Thailand in May 2009. Although influenza A(H1N1)pdm09 quickly became the predominant circulating influenza subtype, circulation of seasonal influenza viruses continued. We used ongoing active, population-based surveillance in two Thailand provinces [7] to describe the incidence and epidemiology of hospitalizations associated with influenza A(H1N1)pdm09 and influenza viruses overall, and to compare characteristics among patients infected with influenza A(H1N1)pdm09 and those infected with other influenza viruses.

## Methods

### Setting

We have conducted active, population-based surveillance for community-acquired pneumonia in all twenty hospitals in two Thailand provinces since 2002–2003 [8]. Sa Kaeo province, on the Cambodian border, had a 2009 population of 544,000, and Nakhon Phanom province, which borders Laos in northeastern Thailand, had a 2009 population of 747,000 [9]. In 2010, among 76 Thai provinces, Nakhon Phanom ranked 72^nd^ with a per capita GDP of $1290 while Sa Kaeo ranked 51^st^ with a per capita GDP of $2,303 [10].

### Patients

A case of acute lower respiratory infection (ALRI) was defined as evidence of both active infection (at least one of: reported fever, reported chills, measured temperature >38.2 or <35°C, or an abnormal white blood cell count or differential) and lower respiratory tract disease (at least one of: abnormal breath sounds, documented tachypnea, cough, sputum production, or dyspnea) in a hospitalized patient. Every other ALRI case-patient was systematically sampled for possible participation in an etiology study. Sampling was done by ward (e.g., male, female, pediatric) and chronologically based on admission time. Chest radiographs, if performed, were digitized and interpreted by a panel of radiologists using standard criteria as previously described [11].

### Ethics Statement

All adult participants and guardians of children aged <18 years provided written consent for study enrollment. A CDC Institutional Review Board and the Ethical Review Committee of the Thailand Ministry of Public Health approved this study.

### Laboratory Testing

Nasopharyngeal swab specimens were collected from enrolled patients using a polyester swab (Puritan®, Guilford, ME, USA). In Nakhon Phanom, nasopharyngeal specimens were collected using flocked swabs (FLOQSwabs™, Copan, Murrieta, CA, USA) starting in July 2010. Specimens were collected at the time of enrollment and were stored at 4–8°C for <24 hours before being frozen at −70°C. Specimens were transported weekly on dry ice to the Thailand National Institute of Health. All specimens were tested for influenza A and B viruses by real-time reverse transcription polymerase chain reaction (rRT-PCR) [12]. Specimens that were positive for influenza A were tested for influenza A subtypes using probes and primers developed by the U.S. CDC for H1 (i.e., seasonal H1), H3, and influenza A(H1N1)pdm09 [12]. Nasopharyngeal specimens were also tested for respiratory syncytial virus and adenovirus by PCR [7]. Participants aged >17 years provided urine that was tested for the presence of *Streptococcus pneumoniae* (pneumococcus) by antigen detection using the Binax NOW® rapid immunochromatographic assay (Scarborough, ME). Blood cultures were performed as clinically indicated [13].

### Supplemental Data Collection

As part of the response to the 2009 influenza pandemic, additional information on underlying conditions, influenza vaccination status, and antiviral treatment was collected using a standard questionnaire on a non-systematic sample of study patients from October 2009 through August 2010.

### Statistical Analysis

The study period included January 2009 through December 2010. Clinical and demographic characteristics were compared among patients infected with influenza A virus subtypes and those infected with influenza B virus. Dichotomous variables were compared by chi-square or Fisher’s exact test and continuous variables by t-test or Kruskal-Wallis. The crude age-specific incidence of influenza-associated ALRI hospitalizations was calculated for the 2-year study period by dividing the number of rRT-PCR-confirmed influenza cases by the combined population of the two provinces [9]. To account for missed cases due to the sampling frame and non-enrollment of eligible patients, we calculated the adjusted incidence by assuming that the proportion positive for influenza viruses was the same among hospitalized, enrolled ALRI patients as hospitalized ALRI patients who did not enroll. The proportion of enrolled patients that were influenza virus positive was multiplied by the total number of eligible (i.e., hospitalized with ALRI) patients to serve as the numerator for adjusted incidence calculations. Patients hospitalized with ALRI >1 times within 14 days were counted as a single case. Confidence intervals (CI) around the adjusted influenza incidence were calculated using the 95% CI for the proportion of influenza positive cases assuming a binomial distribution.

## Results

**Figure 1:**
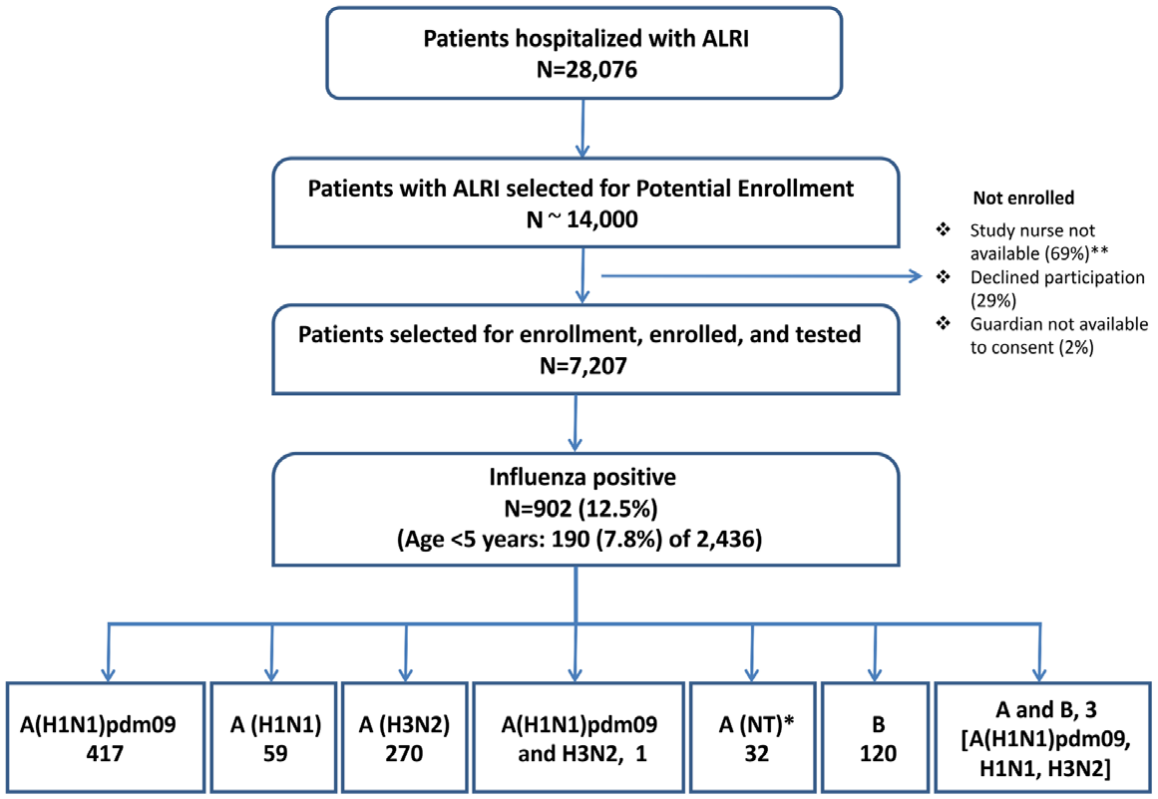
Influenza cases among patients hospitalized with acute lower respiratory infection (ALRI). Detailed legend. Influenza cases among patients hospitalized with acute lower respiratory infection (ALRI) – Sa Kaeo and Nakhon Phanom, Thailand, January 2009 – December 2010. *NT, not typed **Nurses did not routinely enroll patients at night or on weekends.

During the study period, 28,076 patients were hospitalized with ALRI (Figure 1). Of those, approximately 14,000 were sampled for potential enrollment, and 7,207 consented to enroll and were tested for influenza viruses; reasons for non-enrollment included admission during evenings or weekends when study nurses were not available (69%), patient or guardian refusal (29%), and guardian unavailable to consent (2.3%). Among ALRI patients who enrolled and were tested for influenza viruses, 3.1% (225) required mechanical ventilation and 1.5% (110) died compared to 6.9% (1432) and 3.9% (807) of those who did not enroll (p<0.01 for both). These differences persisted across age groups; the difference in case fatality proportion for enrolled vs. non-enrolled was greater for patients aged ≥5 years (2.2% vs. 5.8%) than those <5 years (0.25% vs. 0.38%).

Of the 7,207 enrolled patients, 902 (12.5%) tested positive for an influenza virus, including 190 (7.8%) of 2,436 children aged <5 years. Compared to influenza-negative patients, influenza-positive patients were less likely to be age <5 years (21% vs. 36%) and less likely to be ≥65 years (14% vs. 23%) (p<0.01). After adjusting for age, influenza-positive patients were more likely than influenza-negative patients to have documented fever >38.0°C (56% vs. 37%, p<0.01), but less likely to die (0.44% vs. 1.7%, p = 0.01), require intubation (1.2% vs. 3.4%, p<0.01), need oxygen therapy (21% vs. 34%, p<0.01), or to have chest radiographs consistent with pneumonia (37% vs. 58%, p<0.01). Nasopharyngeal specimens were collected from influenza-positive patients a mean of 0.86 days after admission compared to 1.04 days for influenza-negative patients (p<0.01).

Among 902 influenza cases, 779 (86%) were influenza A, 120 (13%) were influenza B, and three were positive for both influenza A (one each of influenza A(H1N1)pdm09, H1N1, and H3N2) and B. Among influenza A cases, 417 (54%) were influenza A(H1N1)pdm09, 270 (35%) were H3N2, one was A(H1N1)pdm09 and H3N2, 59 (7.6%) were H1N1, and 32 (4.1%) were not subtyped.

Patients infected with influenza A(H1N1)pdm09 virus were similar in age to those with H1N1 and influenza B but younger than those with H3N2 (Table 1). The presence of fever, duration of hospitalization, and the proportion of patients requiring mechanical ventilation and oxygen therapy were similar across influenza types and subtypes. Only four (0.4%) influenza-infected ALRI patients died: one 55 year-old HIV-infected male infected with influenza A(H1N1)pdm09 and three patients with H3N2 (33 year-old female, 75 year-old male, and 75 year-old female). None of the patients who died were reported to have underlying cardiac, renal, liver, or cerebrovascular disease; the 33 year-old female was not pregnant but had history of malignancy. Of 490 (54%) influenza-infected patients with chest radiographs performed, 411 (84%) had a final reading by the radiologist panel, of which 151 (37%) were interpreted as consistent with pneumonia.

**Table 1:** Characteristics of patients hospitalized with influenza-associated acute lower respiratory infection – Sa Kaeo and Nakhon Phanom, Thailand, January 2009 – December 2010. Values represent n (%) unless otherwise stated.

	A(H1N1)pdm09 (N = 417)	A(H1N1) (N = 59)	A(H3N2) (N = 270)	A, unsubtyped (N = 32)	B (N = 120)	p-value
Age, years, median (range)	16 (0–92)	14 (0–82)	45 (0–95)	15.5 (0–80)	15 (0–86)	<0.01^a^
Age group, years						
Age <5	86 (20.6)*	8 (13.6)	60 (22.2)	5 (15.6)	31 (25.8)	<0.01¥
5–17	136 (32.6)	24 (40.7)	30 (11.1)	14 (43.8)	39 (32.5)	
18–49	117 (28.1)	11 (18.6)	56 (20.7)	4 (12.5)	25 (20.8)	
50–64	45 (10.8)	8 (13.6)	59 (21.8)	5 (15.6)	7 (5.8)	
≥65	33 (7.9)	8 (13.6)	65 (24.1)	4 (12.5)	18 (15.0)	
Temperature >38°C	243 (58.4)	36 (61.0)	153 (56.7)	16 (50.0)	57 (47.5)	0.17¥
Fever by report	409 (98.1)	55 (93.2)	266 (98.5)	31 (96.9)	118 (98.3)	0.07¥
Days in hospital, median	3	3	3	3	3	0.71^a^
Death	1 (0.2)	0	3 (1.1)	0	0	0.29¥
Intubation	7 (1.7)	1 (1.7)	2 (0.8%)	0	1 (1.3)	0.70¥
Oxygen therapy	79 (19.0)	14 (23.7)	71 (26.4)	5 (15.6)	20 (16.7)	0.07¥
Co-infections						
RSV	30 (7.2)^b^	0	29 (10.9)	1 (3.1)	8 (6.7)	0.03¥
Adenovirus	2 (0.48)^b^	1 (1.7)	0	0	0	0.20¥
*Streptococcus pneumoniae* (urine antigen testing among age ≥18 years)*	4 (2.1) of 188^d^	0 of 27	8 (4.7) of 170^d^	0 of 13	1 (2.1)of 47	0.38¥
Blood culture positive of those with culture	1 of 121^c^	0 of 23	0 of 92	0 of 9	0 of 35	
CXR with pneumonia (of those with CXR read)	68 (33.7) of 202	9 (31.0) of 29	48 (32.2) of 149	5 (33.3) of 15	20 (35.1) 57	0.97¥

¥ Pearson chi-square test for homogeneity, excluding influenza A cases without subtype.

a Kruskal-Wallis test, excluding influenza A cases without subtype.

* Among those tested for *Streptococcus pneumoniae* by the Binax® urine antigen assay.

b One A(H1N1)pdm09-infected patient was also positive for adenovirus and RSV.

c One patient with *Staphylococcus aureus.*

d One patient positive for both influenza A(H1N1)pdm09 and A(H3N2) tested positive for *Streptococcus pneumoniae.*

CXR, chest x-ray.

Co-infection with RSV occurred in 30 (7.2%) patients with influenza A(H1N1)pdm09, 29 (11%) with H3N2, and 8 (6.7%) with influenza B virus (7.6% of all influenza-infected patients were RSV-positive vs. 9.3% of influenza-negative patients). Of 68 influenza-RSV co-infections, 56% occurred among children aged <5 years with little variation by influenza subtype. No patients co-infected with influenza and RSV died or required mechanical ventilation, and mean hospital stay did not differ significantly between influenza-RSV-co-infected and influenza-only-infected patients (3.9 vs. 3.6 days, p = 0.35). Among 445 influenza-infected patients aged >17 years tested for pneumococcus by urine antigen assay, 12 (2.7%) were positive compared to 140 (4.4%) of influenza-negative patients. One (25%) of four fatalities in influenza-infected patients was co-infected with pneumococcus compared to 11 (2.5%) of 440 non-fatalities (Fisher’s exact p = 0.10). Patients co-infected with pneumococcus were more likely to require oxygen therapy than those who tested negative for pneumococcus (58% vs. 28%, p = 0.04) but were not more likely to need mechanical ventilation; co-infected patients had longer hospital stays (mean 5.0 vs. 3.8 days, p = 0.23), though not significantly so. Three (0.34%) influenza-infected patients tested positive for adenovirus compared to 145 (2.3%) influenza-negative patients. Blood cultures were performed in 282 (31%) of influenza-infected patients, and only one was positive for a potential pathogen (*Staphylococcus aureus* in a 49 year-old female with influenza A(H1N1)pdm09). In comparison, 2336 influenza-negative patients had blood cultured; 146 had a positive blood culture, including seven positive for *S. aureus* and 12 positive for *S. pneumoniae*. Among those with a blood culture, 25% had received antibiotic treatment before culture collection, which is consistent with previous descriptions from these sites [14]. Seven (0.8%) influenza-infected patients were HIV-positive (two with influenza A(H1N1)pdm09 virus, three with other influenza A subtypes (H1N1, H3N2, and untyped), and two with influenza B.

### Seasonality

**Figure 2:**
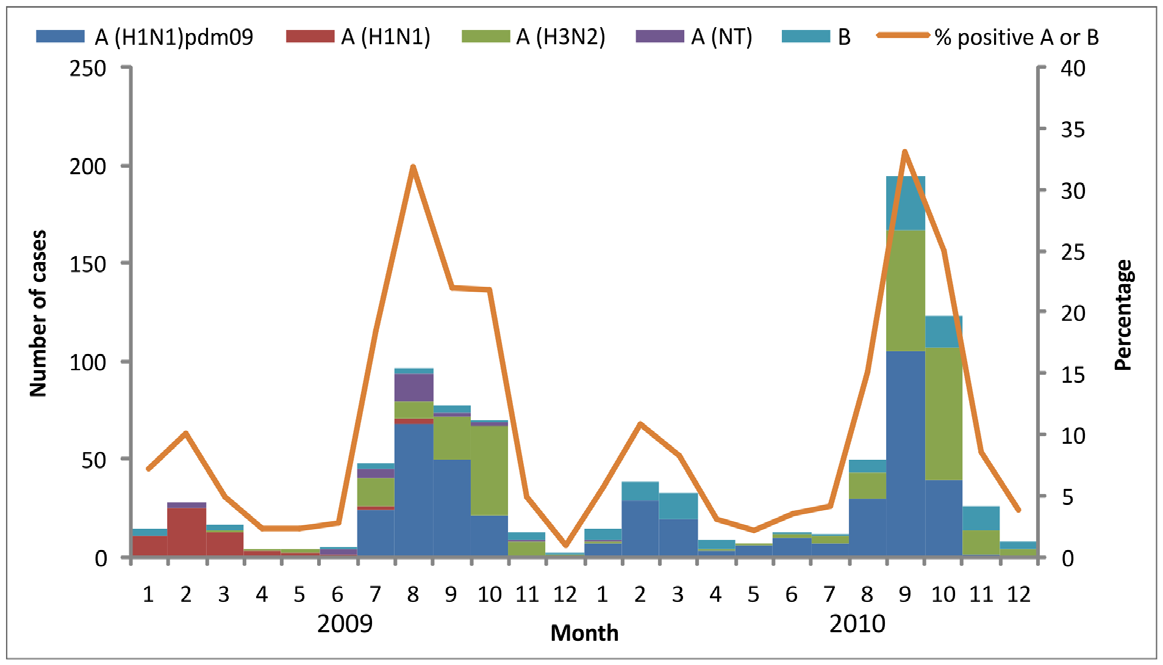
Hospitalized cases of influenza-associated acute lower respiratory infection by month of admission. Detailed legend. Hospitalized cases of influenza-associated acute lower respiratory infection by month of admission – Sa Kaeo and Nakhon Phanom, Thailand, January 2009 – December 2010. Cases with more than one influenza type or subtype are included in each category: three cases positive for influenza B and A (H1N1pdm09, H3N2, H1N1), one case positive for influenza A (H1N1)pdm09 and A (H3N2).

Influenza peaks occurred twice in each study year, with smaller peaks occurring in January – March and larger peaks occurring during July – September (Figure 2). Seasonal influenza A(H1N1) virus predominated in the first peak of 2009 and was infrequent thereafter. Influenza A(H1N1)pdm09 virus predominated in the early part of the second peak in 2009 followed by an increase in H3N2 cases. Influenza infections in 2010 were a mix of influenza A(H1N1)pdm09, H3N2 and influenza B viruses.

### Influenza-associated ALRI Hospitalization Incidence

**Figure 3:**
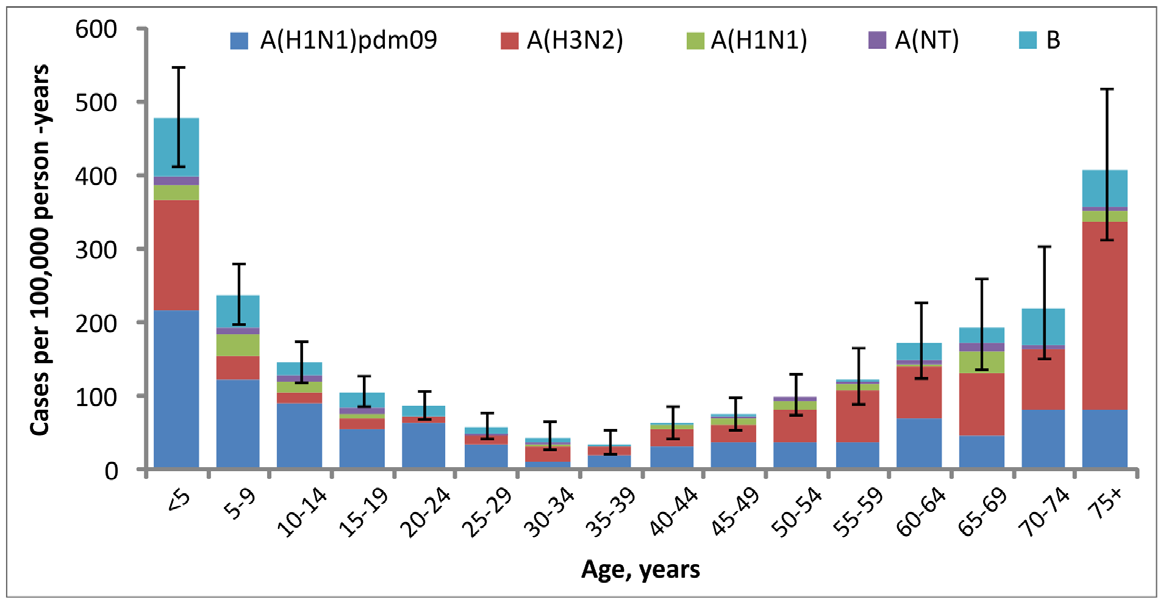
Incidence rate of influenza-associated acute lower respiratory infection hospitalizations. Detailed legend. Incidence rate of influenza-associated acute lower respiratory infection hospitalizations, by type/subtype, Sa Kaeo and Nakhon Phanom provinces, Thailand, January 2009–December 2010. Bars show 95% confidence intervals Rates are adjusted for sampling and non-participation.

The crude incidence rate of influenza-associated ALRI hospitalizations during 2009–2010 was 35 per 100,000 person-years; after adjusting for sampling and enrollment, the overall rate was 136 per 100,000 person-years (95% CI = 127–144 per 100,000). Adjusted rates were highest in children aged <5 years at 477 per 100,000 person-years (95% CI = 414–547 per 100,000) and adults aged ≥75 years at 407 per 100,000 person-years (95% CI = 312–519 per 100,000) (Figure 3). Rates among children aged <1 year did not differ from rates among those <5 years overall. The adjusted incidence rate of hospitalized influenza A(H1N1)pdm09 ALRI cases alone was 63 per 100,000 person-years overall (95% CI = 57–69 per 100,000) (46% of total incidence), 216 per 100,000 among children aged <5 years (95% CI = 173–266 per 10,000) (45% of total), and 83 per 100,000 among adults ≥75 years (95% CI = 43–144 per 10,000) (20% of total). The adjusted influenza-associated hospitalization rate overall in 2010 (143 [95% CI = 131–154] per 100,000 person-years) was slightly higher than that in 2009 (128 [95% CI = 116–141] per 100,000 person-years).

To account for the fact that non-enrolled ALRI patients were more likely to have severe disease (mechanical ventilation or death) than enrolled patients and that a lower proportion of severely ill patients tested positive for influenza viruses, we re-calculated the adjusted incidence using influenza-positive percentages specific to severe vs. non-severe disease. The adjusted incidence accounting for severity was 135 per 100,000 person-years, almost identical to the incidence not accounting for severity (136 per 100,000 person-years).

### Supplemental Data Collection

From October 2009 through August 2010, supplemental data were collected for 475 (14%) study patients, including 31% (82/261) of patients who tested positive for an influenza virus compared to 13% (393/3126) of those who tested negative (p<0.01). Among these 475 patients, 443 (93%) received antiviral treatment (all oseltamivir); 50% received treatment within 2 days of symptom onset and 97% within 2 days of admission. Influenza-negative patients were as likely to receive therapy as those who were influenza-positive (92% vs. 98%, p = 0.09). Influenza vaccination was documented for 32 (7.2%) patients, but only one patient was vaccinated after the monovalent influenza A(H1N1)pdm09 vaccine was available. Co-morbid conditions were identified among 23% of influenza-positive versus 38% of influenza-negative patients (p = 0.01); asthma was most common (16% of influenza-positive).

## Discussion

We documented the substantial incidence and the seasonality of influenza in rural Thailand for a 2-year period that included the 2009 pandemic using ongoing active, population-based surveillance for hospitalized cases of influenza-associated ALRI. Influenza incidence was largely attributable to influenza A(H1N1)pdm09 virus but with important contributions from influenza A(H3N2), and from influenza B. Our results add to the limited but growing literature on the high burden of influenza in tropical settings [1], [3], [15] and highlight the importance of prevention and control measures, which are underutilized in this region where influenza historically has not been considered an important cause of serious illness.

The incidence of hospitalized influenza cases overall was highest in children aged <5 years and adults 75 years and older (>400 per 100,000 person-years in both groups), which is typical of rates documented previously in Thailand [3] and in temperate regions [16]. The incidence in children <5 years (477 per 100,000 person-years) was higher than that in a previous report (120–330 per 100,000) [3], which likely relates to the impact of the pandemic, annual variation in influenza subtypes and associated burden, and a change in enrollment criteria to improve capture of influenza-associated ALRI (eliminated requirement for chest radiograph). The age distribution of influenza A(H1N1)pdm09 cases differed from that of seasonal influenza; the relatively lower incidence in older age groups was consistent with the epidemiology of influenza A(H1N1)pdm09 elsewhere in the world [17] and supports the presence of existing immunity in older populations, as has been documented in the U.S. [18]. Immunity in older age groups was likely due to cross-protective antibodies from infection with related influenza strains to which younger populations had not been exposed [18], [19]. Cross-protective immunity from prior vaccination with related influenza strains is a very unlikely explanation, given the historically low rates of vaccination in Thailand. Although Thailand began providing influenza vaccination to elderly persons in 2009, our data suggest that coverage is low.

Few data on influenza incidence during the pandemic are available from tropical regions. A preliminary report from Guatemala found influenza A(H1N1)pdm09 hospitalization rates of 7.1–8.8 per 100,000 persons during 8 months when circulation was peaking [2]. In the U.S., the CDC’s Emerging Infections Program (EIP) found an influenza hospitalization rate among children aged <5 years during the pandemic (April 2009– May 1, 2010) of 83 per 100,000 [17]; rates in older age groups ranged from 32 to 34 per 100,000. The age-specific rates were substantially lower than our rates for Thailand; for the youngest age groups, rates in Thailand were higher even when limited only to influenza A(H1N1)pdm09, which caused >99% of cases in the U.S. [17]. This comparison is limited by the fact that influenza testing in EIP surveillance was clinician driven, which results in low sensitivity (39% in children aged <5 years [20]) to capture hospitalized influenza cases. However, the sensitivity was likely higher during the pandemic due to more frequent influenza testing and increased use of more sensitive diagnostic tests (i.e., PCR). Even if the U.S. rates in children <5 were adjusted to account for 39% sensitivity (i.e., divided by 0.39), Thailand rates were still higher. A separate analysis of U.S. EIP data corrected for underreporting estimated an adjusted influenza A(H1N1)pdm09 hospitalization rate of 90.2 per 100,000 persons overall (range 64.2–132.4), with the highest rates in ages 0–17 years (117.4 per 100,000, range 83.5–172.4) [21], which are closer to but still lower than Thailand rates.

The co-circulation of seasonal and pandemic influenza viruses during the 2-year period allowed us to compare characteristics of patients infected with different subtypes during the same season. Patients infected with H3N2 virus were significantly older than those infected with influenza A(H1N1)pdm09, H1N1, or influenza B, although the proportion of cases aged <5 years was not significantly different. We found no difference in clinical severity in patients infected with influenza A(H1N1)pdm09 and other influenza viruses. A comparison of patients with pandemic and other influenza viruses in Guatemala found similar characteristics, although patients in the Guatemala study had more severe disease overall [22].

Our findings likely underestimate the rate of severe influenza cases and deaths by 2–3 times, due to challenges enrolling patients with severe illness. ALRI surveillance in these provinces identified 32 deaths among 9,778 children during 2009–10, but only six were enrolled in our study and tested for influenza viruses. An early report of the first 272 hospitalized influenza A(H1N1)pdm09 cases in the U.S. found that 25% required intensive care, 15% needed mechanical ventilation, and 7% died [23]. This compares to only one (0.4%) influenza A(H1N1)pdm09-associated ALRI death in our report (4 (0.4%) influenza deaths overall). The U.S. report may have been biased toward higher severity, because reporting was passive and influenza A(H1N1)pdm09 testing was clinician driven, which favors capturing severe cases. A report from Guatemala among 76 hospitalized influenza A(H1N1)pdm09 cases found that 11% required mechanical ventilation and 15% died [22]. In Malaysia, 3.7% of 1,362 hospitalized patients aged <12 years with influenza A(H1N1)pdm09 died [24]. A case series from another Thailand province described five (21%) deaths among 24 hospitalized adults with influenza A(H1N1)pdm09-associated pneumonia [25]. In contrast, investigators in China actively screened arriving passengers at airports and found no deaths among 426 influenza A(H1N1)pdm09-infected patients, 351 of whom had received oseltamivir [26].

The low case fatality proportion in our sites should be considered in light of the availability and use of oseltamivir in Thailand, where national guidelines during the pandemic recommended treatment of influenza-infected patients with complicated illness (e.g., pneumonia, altered level of consciousness), failure to improve within 48 hours, and high-risk conditions (e.g., pregnancy, chronic illnesses). Data on a subset of our patients showed that >90% of those hospitalized with ALRI received oseltamivir, 50% within two days of symptom onset. Oseltamivir access was likely improved by domestic production of a generic formulation and by prepositioning the drug at government hospitals. Further, hospital access in Thailand is very good, allowing patients to be treated before illness progression. Our lower case fatality proportion may also have been related to the low prevalence of co-morbid conditions (23%) compared to other studies [23], [27]. The relatively low proportion of patients with co-morbid conditions also suggests that clinicians may have had a lower threshold for hospitalization.

Similar to trends seen in the U.S. and other parts of the world, Thailand had a rapid disappearance of the H1N1 virus and relatively lower infection rates from influenza A(H1N1)pdm09 virus in older adults compared to previous influenza seasons. However, in contrast to the U.S. and most temperate regions where influenza A(H1N1)pdm09 accounted for >99% of influenza infections [28], Thailand saw continued circulation of H3N2 and influenza B after the introduction of influenza A(H1N1)pdm09. Influenza peaked in January – March and July – September of 2009 and 2010, consistent with patterns observed in most pre-pandemic years [3], [8]. Surveillance data from 2011 have shown a predominance of influenza A (H3N2) and B in both inpatient and outpatient settings.

Few studies have systematically assessed the prevalence of other pathogens among influenza-infected patients by means other than blood culture [22], [29]. Co-infections with RSV occurred in 7% with the majority among patients aged <5 years and with similar frequency by influenza subtype. We found no differences in clinical severity between influenza-infected patients with and without RSV co-infection. Although numbers were small, our findings suggested that co-infection with pneumococcus increased disease severity, as evidenced by a higher percentage of patients dying and requiring oxygen therapy. Compared to our findings, a report from Guatemala found higher prevalences of co-infection with RSV (12%) and pneumococcus (22%) among hospitalized influenza pneumonia patients [22].

Strengths of our analysis include systematic sampling from active surveillance for ALRI, lack of reliance on clinical reporting or testing, and a known catchment area. However, the findings must be interpreted in light of several limitations. We lacked data on some of the known or potential risk factors for severe influenza infection, including pregnancy and obesity. Our incidence estimates relied on statistical adjustments that assumed enrolled and non-enrolled patients had identical prevalences of influenza positivity. Patients admitted on nights and weekends were under-represented in our sample and may have differed from patients admitted on weekdays in terms of demographics, disease severity, and influenza prevalence. Finally, data on co-infections with bacteria other than pneumococcus relied on blood culture as the single diagnostic modality, and <30% of patients had cultures, 25% of whom were pre-treated with antibiotics.

Using active, population-based surveillance, we found a high incidence of hospitalized influenza ALRI infections in Thailand, exceeding estimates from most other nations and confirming influenza’s substantial contribution to severe respiratory illness, including pneumonia, in Thailand and likely in Southeast Asia. The high disease burden in young children is particularly noteworthy and underscores the role that influenza vaccination should play in strategies to prevent childhood pneumonia globally [30]. During the 2009 pandemic, the Thailand Ministry of Public Health expanded influenza vaccine recommendations to include children age 6 months to 2 years. Efforts to maintain and implement this recommendation are warranted. These data will contribute to ongoing efforts to estimate the global burden of the 2009 influenza pandemic in young children.
